# Comparison of external load during differing microcycle structures across two competitive seasons in elite female Portuguese soccer players

**DOI:** 10.3389/fspor.2025.1608382

**Published:** 2025-06-26

**Authors:** Rafael Oliveira, João Paulo Brito, Renato Fernandes, Mário C. Espada, Fernando J. Santos, Matilde Nalha, Piotr Zmijewski, Ryland Morgans

**Affiliations:** ^1^ Santarém Polytechnic University, School of Sport, Rio Maior, Portugal; ^2^ Life Quality Research Centre (CIEQV), Santarém Polytechnic University, Rio Maior, Portugal; ^3^ Research Center in Sport Sciences, Health Sciences and Human Development (CIDESD), Santarém Polytechnic University, Rio Maior, Portugal; ^4^ Life Quality Research Centre (CIEQV-IP Setúbal), Campus do IPS—Estefanilha, Setúbal, Portugal; ^5^ Escola Superior de Educação, Instituto Politécnico de Setúbal, Setúbal, Portugal; ^6^ Interdisciplinary Centre for the Study of Human Performance (CIPER), Faculdade de Motricidade Humana, Universidade de Lisboa, Lisbon, Portugal; ^7^ SPRINT Sport Physical Activity and Health Research & Innovation Center/Centro de Investigação e Inovação em Desporto Atividade Física e Saúde, Maior, Portugal; ^8^ Comprehensive Health Research Centre (CHRC), Universidade de Évora, Évora, Portugal; ^9^ Faculdade de Motricidade Humana, Universidade de Lisboa, Lisbon, Portugal; ^10^ Jozef Pilsudski University of Physical Education in Warsaw, Warsaw, Poland; ^11^ School of Sport and Health Sciences, Cardiff Metropolitan University, Cardiff, United Kingdom

**Keywords:** GNSS, performance, training, elite European soccer players, training/match load ratio, football

## Abstract

This study aimed to: (i) compare the accumulated load between three and four training sessions per week plus a match across two consecutive seasons in elite female soccer players, and (ii) compare the training/match ratio (TMr) of external load. Data from 10 players in each season were analysed during the study period. The microcycle structure of the first season included three training sessions (3dW) and a match per week, while the second season included four training sessions (4dW) plus a match per week. The following measures were used for analysis: duration, total distance, high-speed running distance (HSR, > 15 km/h), number of accelerations (ACC, > 1–2 m.s^−2^ [ACC1]; > 2–3 m.s^−2^ [ACC2]; > 3–4 m.s^−2^ [ACC3]; > 4 m.s^−2^ [ACC4]) and decelerations (DEC, < 1–2 m.s^−2^ [DEC1]; < 2–3 m.s^−2^ [DEC2];< 3–4 m.s^−2^ [DEC3];< 4 m.s^−2^ [DEC4]). The accumulated load was calculated by summing key metrics for all training sessions and matches, while TMr was calculated by dividing the accumulated load by match data. The main results showed that all variables showed meaningful differences (*p* < 0.05) except for ACC4 and DEC4. Specifically, total distance was higher in 3dW than 4dW (*p* = 0.007), while the remaining variables were higher during 4dW. Moreover, all TMr were higher in 4dW than 3dW (*p* < 0.001 for all variables except for ACC4 and DEC4). As expected, this study showed that adding one training session per week increased accumulated load and TMr for several key variables.

## 1 Introduction

Training and match load quantification in soccer is a routine practice (1–4). Load quantification can be expressed by the locomotor/mechanical demands or physical demands that can associated with external load. Usually, global navigation satellite system (GNSS) metrics, such as distances covered at various running speeds or accelerations, are used to quantify external load (2, 5).

External training load may vary for players according to the number of training/match sessions per week/microcycle due to specific aims of training sessions and the periodisation/planning strategies of the coach (6), or even changing the coach through the season (7). For instance, a recent systematic review in professional male soccer revealed that three to six training sessions were performed per week (8), highlighting that different strategies were applied. Other factors that impact load could be related to the use of specific drills and games during training (e.g., small-sided games, long sprints, repeated sprints, interval training, and medium- to large-sized games) (9). Regarding match-play, load quantification can vary due to the dynamics of matches and contextual factors (10–13).

Previous studies have suggested that the number of training sessions affects the training load in male soccer (14–16). However, it is relevant to mention that different methods for load quantification were used. Anderson et al. analysed the load of each training and match session of three microcycles: a) one with four training sessions and one match; b) another with four training sessions and two matches; and c) with two training sessions and three matches (14). Oliveira et al. analysed the load of each training and match session and the weekly average values of five different microcycles in which all had four training sessions, although the number of matches varied from one to three (15). Nobari et al. analysed the weekly mean values between all microcycles and the accumulated mean values of all weeks included for each microcycle in which the number of training sessions varied from two to six and the number of matches varied from one to two (16).

A method to clarify the understanding of the load induced by training and competitive match-play is to calculate the training/match ratio (TMr) (6, 17, 18). This ratio is calculated by dividing the accumulated weekly load by the match load (6). If the TMr provides a value lower than one, it suggests that the accumulated load of the week is lower than the match load; a value above one suggests that the training load is greater than the match load (6).

Despite the potential practical implications of such analyses, there are a limited number of investigations in male soccer players that examined this ratio with a varying number of training sessions (6, 18) and to the best of authors knowledge, there is no research in female soccer players, although one study analysed the density of training in relation to match-play (19). Clemente et al. compared the TMr of different external load measures between weeks with three, four, and five training sessions/week. The study showed that weeks with five training sessions had higher values for all external load ratios than weeks with three or four training sessions. Additionally, high-speed running (HSR) and sprint distance (SPD) measures presented substantially lower ratios than other variables such as total running distance, accelerations (ACC), decelerations (DEC), and player load (6). Oliveira et al. also compared the TMr of different external load measures between weeks with three, four, five and six training sessions/week. The study showed that weeks with five and six training session presented higher values than weeks with three and four training sessions. The study also demonstrated that weeks with three and four training session had some metrics with TMr values lower than one (e.g, HSR, ACC and DEC) (18). Furthermore, Olaizola et al. compared external load measures of training sessions utilising official match-play as a reference in women's soccer. Key findings revealed that none of the training sessions obtained higher values than matches for all examined measures (19).

Therefore, this study aimed to: (i) compare accumulated and relative accumulated load between three and four training sessions plus a match weeks across two consecutive seasons (2019–20 and 2020–2021) in elite female soccer players, and (ii) to compare the training/match ratio (TMr) of external load. Based on previous studies (6, 18), it was hypothesised that the season with more training sessions will display higher loads.

## 2 Materials and methods

### 2.1 Design

The observational period occurred during two seasons (2019–20 and 2020–2021) of a Portuguese club (from BPI League, the women's first League). The first season, from September to March (early-to-mid-season) was affected by the COVID-19 pandemic, which provoked the disruption of training sessions and matches and the suspension of the season in March. The second season, from September to January, was terminated due to the coach leaving the team. Thus, the observational cohort study contemplated 87 training sessions plus 15 matches for analysis from the 2019-2020 in-season and 100 training sessions plus 10 matches from the 2020-2021 season. The coach and performance staff were the same in both study seasons. In the first season, the typical microcycle had three training sessions and one match per week (MD-5, MD-4, MD-2 and MD) while in the second season, the typical microcycle had four training sessions and one match per week (MD-5, MD-4, MD-3, MD-2 and MD). No gym sessions were included during either season.

### 2.2 Participants

Twenty professional outfield soccer players from a Portuguese club were involved in the study. Data from the complete 2019–20 and 2020–21 seasons included 20 players, 10 in each season (age 24.7 ± 2.4 years, weight 57.9 ± 8.5 kg, height 1.63 ± 0.09 m, body mass index 21.81 ± 3.03 kg/m^2^). The inclusion/exclusion criteria was adopted from a previous study (18) where participants need to achieve a minimum of 80% of the training sessions and a minimum of 45 min of the weekly match, while the exclusion criteria were based on becoming injured, ill, sick for two or more consecutive weeks, player joining the team late in either of the study seasons, lack of full, complete data for training or match-play, and goalkeepers, due to the different variations in the physical demands with outfield players.

All data collected resulted from normal analytical procedures regarding player monitoring over the competitive season, nevertheless, written informed consent was obtained from all participants. Prior to data collection, the club, coaches, and participants were fully informed of the study design and signed an informed consent form. The study was conducted according to the requirements of the Declaration of Helsinki and was approved by the local Research Ethics Committee of the Polytechnic Institute of Santarém, Santarém, Portugal (No. 252020Desporto), and the Portuguese club from which the participants volunteered (20). To ensure confidentiality, all data were anonymised prior to analysis.

Players were assigned to one of three positions as match demands for these differ significantly. The methodology of differentiating specialised positions was adapted from previous research (21). As various situational factors influence the style of play that can be modulated by different tactical roles (10), context was considered whilst using a player's average position in an attempt to determine a player's relevant tactical role in the team (22). Thus, regular playing position was defined at the start of each season, and it remained consistent throughout the study period. Consequently, positions were defined as: defenders (DF, *n* = 5), midfielders (MF; *n* = 9), and strikers (ST; *n* = 6). Goalkeepers were excluded from the investigation due to the specific nature of their match activity and their low running demands (23, 24).

### 2.3 Data collection

A portable 10 Hz GNSS device was used to collect external data (PlayerTek, Catapult Innovations, Melbourne, Australia), which also incorporates a tri-axial 100 Hz accelerometer. These types of GNSS devices seem to be the most valid and reliable to use in team sports (25). Ten minutes before each training session and match, PlayerTek devices were turned on. The devices were turned on and placed in a specific customized vest pocket located on the posterior side of the upper torso fitted tightly to the body, as is typically used in matches. The devices were placed and checked by the same coach, and the players always used the same device (26). The measures used for analysis were duration, total distance, HSR distance (>15 km.h^−1^) (27), number of accelerations (ACC, >1–2 m.s^−2^ [ACC1]; >2–3 m.s^−2^ [ACC2]; >3–4 m.s^−2^ [ACC3]; >4 m.s^−2^ [ACC4]) and decelerations (DEC, <1–2 m.s^−2^ [DEC1]; <2–3 m.s^−2^ [DEC2];<3–4 m.s^−2^ [DEC3];< 4 m.s^−2^ [DEC4]).

### 2.4 Accumulated load and training/match ratios

Absolute accumulated load consisted of the sum of each measure during all training sessions of the microcycle and was calculated per player, thus providing the weekly load for each measure (match included) (6, 18, 28–30). Moreover, the accumulated load was calculated without match data to determine the TMrs for all external measures. Ratios were then calculated by dividing accumulated load (without match data) by match data (TMr = weekly load/match demands) (6, 17, 18, 29). Consequently, the following measures were obtained: total distance ratio (TDr), HSR distance ratio (HSRr), ACC1 ratio (ACC1r), ACC2 ratio (ACC2r), ACC3 ratio (ACC3r), ACC4 ratio (ACC4r), DEC1 ratio (DEC1r), DEC2 ratio (DEC2r), DEC3 ratio (DEC3r), DEC4 ratio (DEC4r). All TMr calculations of load and duration measures provided clear descriptions of the microcycle structures applied. Finally, the relative accumulated load was calculated for each measure by diving absolute accumulated load per absolute accumulated duration of the microcylce.

### 2.5 Statistical analyses

The IBM SPSS Statistics for Windows (version 27.0, IBM Corp, Armonk, NY, USA) was used for all descriptive and inferential statistics. Means ± standard deviations were used for descriptive statistics. All measures were tested for normality and homogeneity using the Shapiro-wilk and Levene tests, respectively. Normal distribution was not confirmed, and thus, non-parametric Mann–Whitney *U*-test was used for the comparisons. A *p* < 0.05 was considered as a significant result. Furthermore, the effect-size (ES) was determined by Hedges' g (by the difference of two means divided by the standard deviation of the different measures). Finally, the ES was interpreted as follows: <0.2 = trivial, 0.2–0.59 = small, 0.6–1.1 = moderate effect, 1.2–2.0 = large effect, >2.0 = very large (31).

## 3 Results

Table 1 presents the comparisons for accumulated load demands (match data included). Except for ACC4 and DEC4, all variables showed meaningful differences (*p* < 0.05) with moderate to very large effect sizes.

**Table 1:** Comparisons of different microcycles for accumulated load demands.

Variable	3dW	4dW	*p*-value	Effect size
Duration (min)	333.26 ± 17.93	441.77 ± 63.72	**0**.**002**	**2**.**22**
Total distance (m)	21,233.74 ± 1,507.63	15,284.03 ± 4,324.61	**0**.**007**	**1**.**76**
HSR (m)	1,810.35 ± 591.18	4,044.77 ± 1,384.47	**<0**.**001**	**1**.**62**
ACC1 (nr)	551.30 ± 66.74	651.52 ± 108.64	**0**.**009**	**0**.**93**
ACC2 (nr)	320.50 ± 40.03	428.46 ± 69.80	**<0**.**001**	**1**.**56**
ACC3 (nr)	112.83 ± 29.57	149.76 ± 30.11	**0**.**011**	**1**.**23**
ACC4 (nr)	37.15 ± 16.27	49.59 ± 30.77	0.280	-
DEC1 (nr)	506.70 ± 55.65	634.83 ± 105.86	**0**.**007**	**1**.**22**
DEC2 (nr)	310.05 ± 43.80	395.69 ± 62.24	**0**.**002**	**1**.**39**
DEC3 (nr)	116.20 ± 29.68	157.34 ± 45.49	**0**.**035**	**0**.**92**
DEC4 (nr)	51.74 ± 21.25	83.16 ± 58.59	0.063	-

3dW, three days week microcycle; 4dW, four days week microcycle; m: meters; min: minutes; HSR: high-speed running (>15 km/h); ACC1, acceleration 1; ACC2, acceleration 2; ACC3, acceleration 3; ACC4, acceleration 4; DEC1, deceleration 1; DEC2, deceleration 2; DEC3, deceleration 3; DEC4, deceleration 4; bold: significant results.

Table 2 presents comparisons for all TMr. Apart for ACC4r and DEC4r, all variables showed meaningful differences (*p* < 0.001) with moderate to very large effect sizes and only ACC3r showed a small effect.

**Table 2:** Comparisons of the different microcycles for TMr.

Variable	3dW	4dW	*p*-value	Effect size
TDr (A.U.)	1.84 ± 0.36	3.99 ± 1.70	**<0**.**001**	**3**.**25**
HSRr (A.U.)	1.11 ± 0.27	1.94 ± 0.64	**<0**.**001**	**2**.**73**
ACC1r (A.U.)	2.15 ± 0.45	3.63 ± 1.20	**<0**.**001**	**2**.**33**
ACC2r (A.U.)	2.06 ± 0.49	3.81 ± 1.14	**<0**.**001**	**2**.**72**
ACC3r (A.U.)	2.30 ± 0.46	4.65 ± 1.25	**<0**.**001**	**0**.**20**
ACC4r (A.U.)	2.79 ± 0.56	6.20 ± 5.81	0.796	-
DEC1r (A.U.)	2.01 ± 0.33	3.49 ± 1.09	**<0**.**001**	**0**.**95**
DEC2r (A.U.)	2.19 ± 0.45	4.17 ± 1.28	**<0**.**001**	**3**.**00**
DEC3r (A.U.)	2.11 ± 0.59	3.99 ± 1.38	**<0**.**001**	**2**.**41**
DEC4r (A.U.)	1.92 ± 0.72	3.86 ± 2.60	0.063	-

3dW, three days week microcycle; 4dW, four days week microcycle; A.U., arbitrary units; TDr, total distance ratio; HSRr: high-speed running ratio (>15 km/h); ACC1r, acceleration 1 ratio; ACC2r, acceleration 2 ratio; ACC3r, acceleration 3 ratio; ACC4r, acceleration 4 ratio; DEC1r, deceleration 1 ratio; DEC2r, deceleration 2 ratio; DEC3r, deceleration 3 ratio; DEC4r, deceleration 4 ratio; bold: significant results.

**Figure 1:**
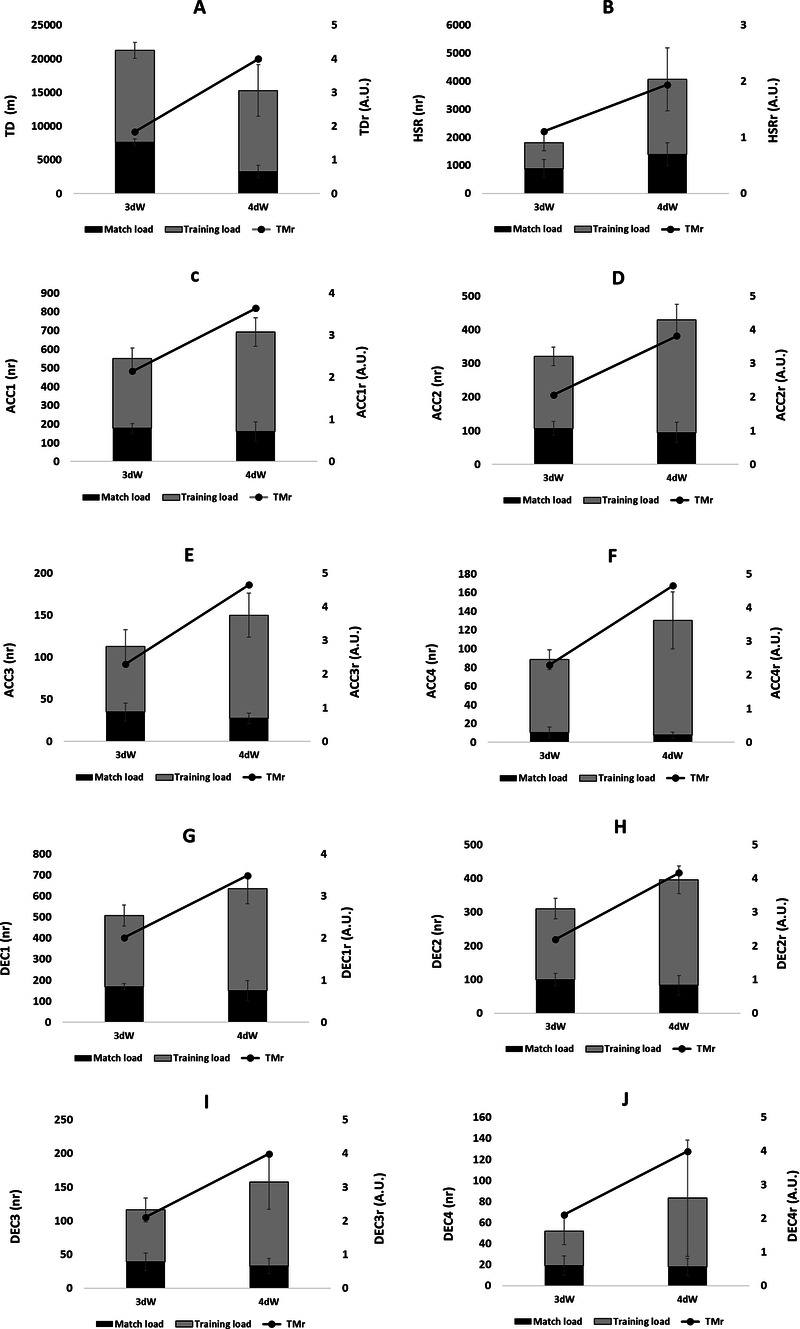
Accumulative weekly training load, match load and training/match ratios. **(A)** TD; **(B)** HSR; **(C)** ACC1; **(D)** ACC2; **(E)** ACC3; **(F)** ACC4; **(G)** DEC1; **(H)** DEC2; **(I)** DEC3; **(J)** DEC4.

Figure 1 shows the accumulative weekly training load, match load and training/match ratios per microcycle.

**Figure 2:**
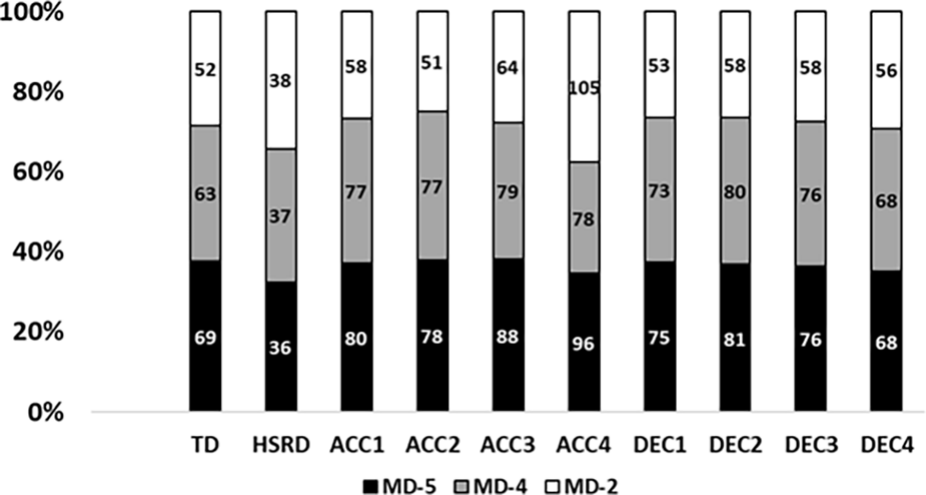
Percentages per each training day of all metrics in 3dW. MD-5, match-day minus 5; MD-4, match-day minus 4; MD-3, match-day minus 3.

**Figure 3:**
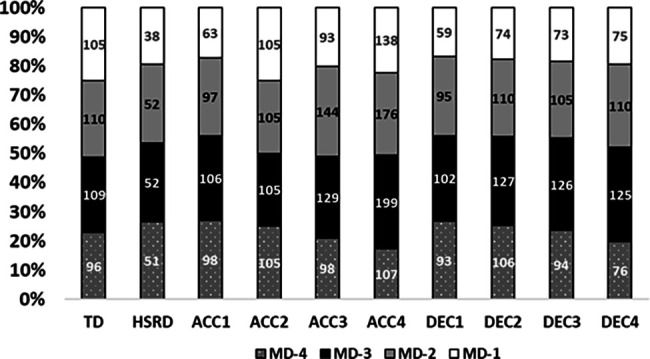
Percentages per each training day of all metrics in 4dW. MD-4, match-day minus 4; MD-3, match-day minus 3; MD-2, match-day minus 2; MD-1, match-day minus 1.

Figure 2 shows match-day minus description of the different metrics in percentages of the 3dW, while Figure 3 presents 4dW.

Table 3 presents comparisons for relative accumulated load demands. Only total distance and HSR showed meaningful differences (*p* < 0.05) with large to very large effect sizes.

**Table 3:** Comparisons of different microcycles for relative accumulated load demands.

Variable	3dW	4dW	*p*-value	Effect size
Total distance (m/min)	63.80 ± 4.62	35.17 ± 11.97	**<0**.**001**	**4.19**
HSR (m/min)	5.46 ± 1.86	9.27 ± 3.15	**0**.**007**	**1.61**
ACC1 (nr/min)	1.65 ± 0.16	1.58 ± 0.20	0.353	**-**
ACC2 (nr/min)	0.96 ± 0.12	0.98 ± 0.17	>0.999	-
ACC3 (nr/min)	0.34 ± 0.09	0.35 ± 0.09	0.684	**-**
ACC4 (nr/min)	0.11 ± 0.05	0.12 ± 0.07	0.796	-
DEC1 (nr/min)	1.52 ± 0.16	1.45 ± 0.20	0.393	-
DEC2 (nr/min)	0.93 ± 0.13	0.91 ± 0.16	0.529	-
DEC3 (nr/min)	0.35 ± 0.09	0.36 ± 0.12	0.796	-
DEC4 (nr/min)	0.16 ± 0.07	0.19 ± 0.12	0.739	-

3dW, three days week microcycle; 4dW, four days week microcycle; m: meters; min: minutes; HSR: high-speed running (>15 km/h); ACC1, acceleration 1; ACC2, acceleration 2; ACC3, acceleration 3; ACC4, acceleration 4; DEC1, deceleration 1; DEC2, deceleration 2; DEC3, deceleration 3; DEC4, deceleration 4; bold: significant results.

## 4 Discussion

The aims of this study were to: (i) compare accumulated and relative accumulated load between three and four training session plus a match across two consecutive seasons (2019–20 and 2020–2021) in elite female soccer players, and (ii) compare the training/match ratio (TMr) of external load.

Training load monitoring has become a relevant aspect between research and practice to control training and match demands in team sports such as soccer. Comparing accumulated training and match load in weeks with varying numbers of training sessions can help support the understanding of whether an increase in the different load metrics is evident (32). The use of GNSS technology provides valuable information on external load in terms of the volume and intensity of each training session and match (33). Previous research has shown that over or under exposure to some external loading metrics, such as HSR and ACC/DEC, may increase the risk of non-contact injuries (34, 35). Similarly, higher accumulated total distance has been associated with injury in female soccer players (36).

The present study findings revealed that when comparing accumulated load demands between three and four training sessions across two consecutive seasons (match data included) all variables, except for ACC >4 m.s^−2^ and DEC <4 m.s^−2^, showed meaningful differences (*p* < 0.05) with moderate to very large effect sizes.

Previous research suggested that match-play represents the greatest physiological stimulus and represents the primary performance outcome (37, 38). Nonetheless, nearly 80% of the weekly training load resulted from the training sessions, whereas about 20% came from the match-play (39, 40). Understanding the cumulative effect of training is essential to guide the individual athlete's performance (5, 41). To optimise athletic performance, training sessions should be prescribed to suit an individual athlete's physical characteristics. However, in team sports this is complex by nature and complicated to apply (42). In the Portuguese female soccer league, training sessions are often conducted three times per week, which reduces the likelihood that players are performing training appropriate to the demands of the game (43). Quantification of training/match load represents an important procedure to adjust the training stimuli provided to players to adequately prepare for match demands (5, 6, 18).

The differences in external load between microcycles with three and four training sessions plus a match in elite female soccer players across two competitive seasons can potentially be partly explained by several factors, including training periodization, recovery strategies, match intensity, and player fitness levels. When analysing the average duration of the training sessions plus the match, in the two competitive microcycles of three and four sessions, the values ​​were similar, 83.15 and 88.34 min, respectively. Regardless of the number of weekly sessions, the accumulated duration appears to maintain the same temporal pattern. For total distance, there was a significant decrease in the distance covered in the four-sessions microcycle. This may possibly be due to the coach opting for a weekly training structure with more sessions dedicated to tactical and skills training, although the analysis of the training content was beyond the scope of this research, in the season with four weekly training sessions. However, the total distance covered in the microcycles with three sessions is similar to those in one of the few studies that analysed training load in women's soccer (44). As reported previously, during a competitive microcycle, the workload may be influenced by contextual factors such as the length of the microcycle (45, 46). Furthermore, Gualtieri et al. established that practitioners adjusted the microcycle schedule based on specific microcycle lengths (i.e., 3-, 4-, 5-days), reducing muscular impact (i.e., ACC and DEC) and using the 5-day microcycle as an opportunity for recovery (47). However, in the present study, the absolute accumulated load of ACC and DEC was higher in the 4dW. When comparing the number of ACC between microcycles, the 4dW reported higher values, except for ACC4. The same pattern was observed for DEC. Posse-Álvarez et al. reported that longer microcycles (four or five days) showed significantly greater ACC and DEC than shorter microcycles (two days) (46). Perhaps these differences in mechanical load could be related to shorter training session durations in the four-session microcycle (∼15 min less than the three-session week).

It is important to note that in the present study, the match number was higher in the three-session microcycle (15 vs. 10 matches), which may have contributed to the differences in total distance between the microcycles. Despite using the average values for analysis, there was a higher standard deviation for the total distance metric in the second season than in the first. Other justifications may be associated with the highest intensity displayed in the 4dW while all other metrics were higher when compared with 3dW. In other words, the higher intensity and number of matches performed in the second season (4dW) contributed to a lower total distance covered when compared with the first season (3dW). Additionally, the increase in the number of training days and the loading pattern of the 4dW compared with the 3dW may further affect physical performance during match-play during the second season. For example, the present study did not examine the differences in training sessions (e.g., MD-5, MD-4, MD-3, MD-2) which may explain the lower total distances in the second season, while sprint distance (e.g., > 18 km.h^−1^) that is known to impact match-play (48, 49), was not reported, since the present study used a different threshold (>15 km.h^−1^) of HSR (27).

The distances covered at HSR in the two microcycles were significantly different, with a greater distance covered in the 4dW (1820.35 vs. 4,044.77 m). However, teams often manage loads by adjusting intensity in 4dW to avoid excessive fatigue before matches (50). The 3dW often allow enhanced recovery, potentially benefiting match performance, especially in congested periods. In the study by Mara et al., a typical weekly in-season training structure consisted of one match followed by two recovery days, one conditioning session, one skill session, and two tactical sessions, with a distance of 1,027 m reported during the early season (44). However, other authors reported that female players covered 718 m, which was around ∼7% of the match total distance (51). However, attention should be given to HSR and SPD distances as important indicators of match physical performance (37, 51).

Overall, this study demonstrated that the external load of training microcycles varies depending on the length of the microcycles in professional female soccer, and these results are also supported by a recent study (46). Considering that the quantification of training/match load represents an important procedure for adjusting training stimuli provided to players to adequately cope with the demands of the match (33, 38), in the present study, the training/match ratios were analysed. Thus, from the analysis of the TMr, although the relationship between accumulated weekly training load and match demands varies according to the nature of the external load measure, it was observed that all metrics showed meaningful differences, except for ACC4 and DEC4 (without significant results). Oliva-Lozano et al. reported that the length of the microcycle had a significant effect on the load, not only in the volume but also in the intensity, except for relatively high ACC and DEC (45). This study found that the TMr were higher in the 4dW, while previous studies showed that the length of the microcycle may have an impact on the workload of soccer players (6, 14–16, 18, 33, 52), which supports the present findings. As an interpretation guidance, when the TMr is higher than one, it suggests that the weekly training load is greater than the match load. When the value is lower than one, it reports that the weekly training load is lower than the match load. When the value is equal to one, it means that both are equivalent (6). In the present study, only HSRr in the 3dW presented a close value to one (1.11 A.U.), suggesting that HSR was slightly higher than during a match.

Finally, the relative accumulated load calculated for each measure (absolute accumulated load/absolute accumulated duration of the microcycle) showed significant differences, although only in total distance (63.80 vs. 35.17 m/min) and HSR distance (5.46 vs. 9.27 m/min). The results from recent studies have shown that the training load increased with longer microcycles (6, 18, 53). This is a questionable issue, as our training load data did not consistently show this tendency, which could be attributed to variations in the training methodologies used by the coaches (45).

This study addressed a general overview of load monitoring in professional female soccer players. Considering that the training load can be influenced by the type of microcyle, player's starting status, playing positions, training mode, and other contextual factors, the comparison of the two seasons indicates that accumulated load and TMr increase with the number of weekly training sessions. Understanding seasonal competitive training/match load variations and the relationships between measures seems important to define the most appropriate monitoring strategy of external load during varying microcycle structures.

This study is not without limitations that could inform future research. For instance, it analysed only a single professional female soccer team from a Portuguese league, thus highlighting the potential value of conducting a multi-club study across different leagues and countries. Still, caution should be taken when interpreting the results. Additionally, future studies could incorporate internal load variables (e.g., RPE, mean heart rate) and contextual variables such as match result, opponents, and location since they have been shown to play some role in load distribution across weeks in female players (54).

## 5 Conclusion

This study showed that adding one training session per week increased accumulated load and TMr for several key variables, which suggest that in the first season, additional load may have been applied even in fewer training sessions. Specifically, when analysing accumulated load demands, more total distance was covered in 3dW than 4dW, while all others were higher in 4dW. Moreover, when considering TMr, all metrics were higher in 4dW. Still, no TMr lower than 1.0 were found. Lastly, when considering relative accumulated load demands, more total distance was covered in 3dW than 4dW while more HSR was covered in 4dW than 3dW. However, no other meaningful differences were found which reveals the importance on how analysing data can change the results interpretation.

## Data Availability

The raw data supporting the conclusions of this article will be made available by the authors, without undue reservation.
